# Intermittent C1-Inhibitor Deficiency Associated with Recessive Inheritance: Functional and Structural Insight

**DOI:** 10.1038/s41598-017-16667-w

**Published:** 2018-01-17

**Authors:** Sonia Caccia, Chiara Suffritti, Thomas Carzaniga, Romina Berardelli, Silvia Berra, Vincenzo Martorana, Annamaria Fra, Christian Drouet, Marco Cicardi

**Affiliations:** 1 “L. Sacco” Department of Biomedical and Clinical Sciences, University of Milan, via GB Grassi 74, 20157 Milan, Italy; 2 Department of Molecular and Translational Medicine, University of Brescia, Brescia, Italy; 3 Institute of Biophysics, National Research Council of Italy, Palermo, Italy; 4 GREPI EA7408, Universite Grenoble Alpes, and CREAK, CHU Grenoble, Grenoble, France; 5 Luigi Sacco Hospital, Milan, Italy

## Abstract

C1-inhibitor is a serine protease inhibitor (serpin) controlling complement and contact system activation. Gene mutations result in reduced C1-inhibitor functional plasma level causing hereditary angioedema, a life-threatening disorder. Despite a stable defect, the clinical expression of hereditary angioedema is unpredictable, and the molecular mechanism underlying this variability remains undisclosed. Here we report functional and structural studies on the Arg378Cys C1-inhibitor mutant found in a patient presenting reduced C1-inhibitor levels, episodically undergoing normalization. Expression studies resulted in a drop in mutant C1-innhibitor secretion compared to wild-type. Notwithstanding, the purified proteins had similar features. Thermal denaturation experiments showed a comparable denaturation profile, but the mutant thermal stability decays when tested in conditions reproducing intracellular crowding.Our findings suggest that once correctly folded, the Arg378Cys C1-inhibitor is secreted as an active, although quite unstable, monomer. However, it could bear a folding defect, occasionally promoting protein oligomerization and interfering with the secretion process, thus accounting for its plasma level variability. This defect is exacerbated by the nature of the mutation since the acquired cysteine leads to the formation of non-functional homodimers through inter-molecular disulphide bonding. All the proposed phenomena could be modulated by specific environmental conditions, rendering this mutant exceptionally vulnerable to mild stress.

## Introduction

C1-inhibitor (C1-INH) (UniProt ID: P05155) is a protease inhibitor of the serpin (Serine Protease Inhibitor) superfamily, controlling complement and kinin/contact system activation^1^. Its genetic deficiency leads to the most common form of hereditary angioedema, which is usually transmitted as an autosomal dominant character (C1-INH-HAE) (OMIM ID: 106100)^2^. The clinical phenotype manifests with disabling and potentially lethal angioedema, i.e. episodic local edema of the subcutaneous and submucosal tissues, when one of the two alleles of C1-INH gene (*SERPING1*) is mutated^3,4^. Several hundred mutations in *SERPING1* have been associated with C1-INH-HAE (HAEdb, http://hae.enzim.hu)^5^, with the same mutation rarely found in unrelated families^6–8^. Genetic variability results in two variants of C1-INH-HAE, type 1 and type 2. In their original description type 1 “appears to result from failure to synthesize the esterase inhibitor”, whereas in type 2 “an abnormal, non-functional protein is synthesized”^9^. While reported mutations leading to type 1 are dispersed throughout the entire *SERPING1*, type 2 mutations map around the protein reactive centre loop (RCL), with the single exception of a mutation in the amino acid residue Lys251, which is far from the RCL, but affects its function after protein folding^10^. Despite the abundance of genetic differences, the clinical variability only affects the frequency and severity of symptoms and does not correlate with the genotype^11^. This is true also in the few patients - nine in five independent families - described to carry homozygous C1-INH deficiency. Their clinical phenotype is similar to that of heterozygous deficiencies. However, in four of the five families, only homozygous subjects manifested angioedema symptoms suggesting a recessive trait^12–15^. In the fourth family the index case was a *de novo* mutation and no heterozygous subjects were present^16^.

**Figure 1:**
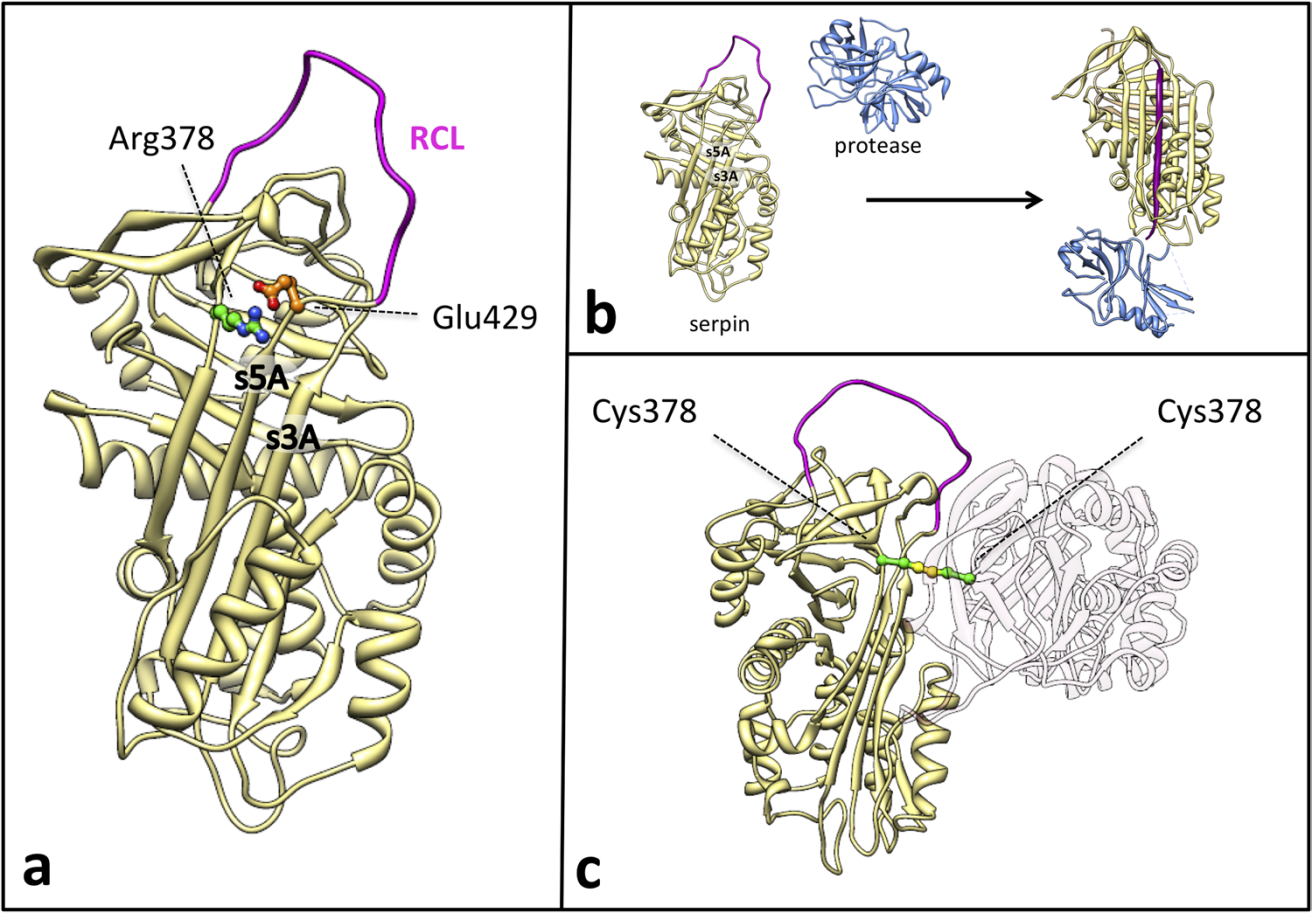
(**a**) Structural localization of the Arg378Cys missense variants in the C1-INH serpin domain. Active inhibitory serpins in their native state are characterized by a central β-sheet (β-sheet A) and an exposed Reactive Centre Loop (RCL in purple) presenting the bite for the target protease. Arg378 (depicted as ball & stick, with C atoms in green and N atoms in blue), at the top of the β-sheet A, is expected to form a salt bridge with Glu429 (depicted as ball & stick, with C atoms in orange and O atoms in red) in the hinge of the RCL, that may regulate the opening of the β-sheet A at the insertion of the RCL between strand 3 (s3A) and strand 5 (s5A). (**b**) *Serpins inhibitory mechanism*. After the docking of the free protease (blue) and the active serpin (khaki), the protease cleaves the RCL (purple). RCL cleavage triggers a profound conformational change within the serpin molecule: the RCL downstream of the scissile bond inserts between strands 3 and 5 of β-sheet A as strand 4 while the protease is translocated and crushed against the opposite pole of the inhibitor molecule, resulting in enzyme inhibition by active site distortion. This conformational rearrangement greatly augments the stability of the serpin molecule. Insertion of the RCL in the structural core of the serpin can also happen without RCL cleavage (latentisation or polymerisation process) (**c**) *Model of the disulfide-linked dimer of mutated C1-INH*. Two mutated C1-INH molecules (khaki and light pink) interact in a dimer. The two Cys at position 378 (depicted as ball & stick, with C atoms in green and S atoms in yellow) form a disulphide bridge (highlighted in yellow) that covalently bounds the two molecules. The second molecule has been drawn nearly transparent for clarity purposes. We must consider that the disulphide bridge does not necessarily occur between native-like structures as reported in the picture. The coordinates used are from pdb entry **1M6Q** for C1-INH as active serpin (homology model of the native C1-INH serpin domain), **1K1L** for free protease, and **1EZY** for serpin-protease complex. Figures were produced using Chimera^72^.

Serpin inhibitory function requires a notable change in protein topology (Fig. 1b), which largely increases the stability of the serpin molecule^17^. The same molecular flexibility and propensity to transition to a more stable conformation renders serpins extremely vulnerable to missense mutations. A single amino acid change can cause such a sophisticated mechanism of action to crumble (loss of function), or could render serpins susceptible to misfolding, often associated to a variable degree of protein polymerization (gain of function)^18,19^.

Misfolding and polymerization of mutated serpins are at the base of a group of conformational diseases collectively known as serpinopathies^18,20^. Likewise, missense mutations in *SERPING1* can cause polymerization and impaired protein secretion that lead to C1-INH deficiency and C1-INH-HAE^21–24^. Nevertheless, none of these mutations has been correlated to a specific clinical phenotype.

It is widely recognized that *SERPING1* mutations cannot predict C1-INH-HAE phenotype. This conclusion is drawn on lack of significant evidence based on studies comprehensively addressing the question.

Here we studied a mutant C1-INH in which Arg378 (C1-INH mature protein numbering) located at the top of β-sheet A is substituted with Cys (Fig. 1a). This is a naturally occurring mutant that was found in a subject presenting with profound, intermittent C1-INH deficiency and absence of typical HAE phenotype. The same mutation has been previously reported in homozygous form to be responsible for recessive HAE^14^. We addressed the hypothesis that this mutation belongs to the class of “conformational” mutations and under mild stress conditions prompts protein misfolding with both loss and gain of function. Indeed, in particularly challenging environments, such as the crowded intracellular environment, where C1-INH concentrations are high, the mutated protein could oligomerize, also recruiting wild-type monomers into the growing polymers, and erratically impairing total protein secretion from cells. Such impairment may result in variability of C1-INH plasma levels that require a homozygous state to express a HAE phenotype. To evaluate this hypothesis, we regularly collected plasma samples for biochemical analysis and ran cellular, structural and functional studies on recombinant proteins.

## Results

### Brief case report

Patient T.M. is a 64-year old woman who came to our observation in October 1996 for recurrent abdominal pain, generalized pruritus and low levels of C4. The symptoms started when she was 21-year old and consisted in diffuse abdominal crampy pain, nausea, but no vomit. Most of the times these symptoms subsided spontaneously in 4–6 hours; in a few instances there was need for spasmolytics, butylscopalamine, or for the non oppiaceous pain killer, tramadol. The frequency of symptoms varied during life alternating recurrences every other day to several months completely symptom-free. The patient reported appearance of abdominal symptoms after ingestion of specific foods (shellfish, tomatoes, cheese), but the majority of recurrences were without apparent reason. She never experienced symptoms referable to angioedema of the skin, oral cavity or larynx. Family history was negative for any angioedema symptom. No ancestor was available for testing; her two daughters had normal C1-INH plasma levels and were negative for mutations in *SERPING*1. Because of the evidence of C1-INH deficiency and presence of abdominal pain recurrences, the patient was treated with danazol. She remained on this therapy intermittently until she decided to stop it because of side effects of attenuated androgens.

### Complement parameters

Complement parameters were measured at different time points over the years (supplementary Figure 1). C1-INH antigen and function, as well as C4, range from extremely low values that are typical of C1-INH-HAE to completely normal values. C1-INH antigen was almost always slightly higher than function.

### *SERPING*1 gene sequencing and expression

Sequencing of *SERPING1* demonstrated that patient T.M. is heterozygous for the nucleotide substitution NG_009625.1:g.19332 C > T (rs201363394), which leads to the amino acid mutation Arg378Cys (mature C1-INH numbering). This missense mutation is situated in exon 7. Residue 378 is located in the serpin domain, at the top of the central β-sheet and Arg378 is expected to form a salt bridge with Glu429 (Fig. 1a). This position is occupied by an electropositive residue (Lys 72%, Arg 23%) in 208 serpin sequences out of 219^25^.

Real-time reverse transcription polymerase chain reaction (RT-PCR) showed no impairment in C1-INH mRNA levels in peripheral blood mononuclear cell from patient T.M. compared to healthy donors (data not shown).

### Effects of the Arg378Cys mutation on plasma C1-INH

**Figure 2:**
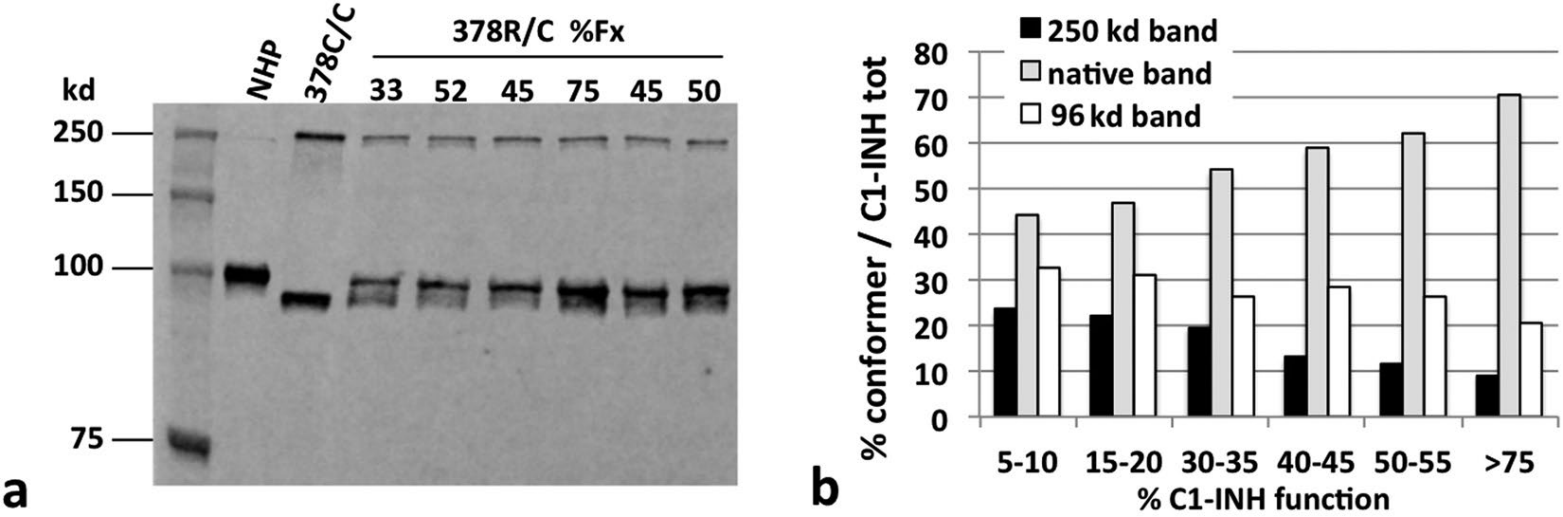
Plasma C1-INH conformers versus function. (**a**) Non-reducing SDS-PAGE and immunoblot analysis of plasma from patient T.M. (378 R/C) at different functional values of C1-INH, showing the native (105 kd), and the aberrant conformers of 96 kd and 250 kd. Equal plasma volumes were loaded in each lane. Normal human plasma (NHP) and the Arg378Cys homozygote plasma (378C/C) are shown as reference. The primary Ab was the 12-27-15 monoclonal antibody, revealed with a secondary Ab designed for fluorescent detection. The figure represents part of the samples evaluated in panel b. (**b**) The relative amount of the different conformers is plotted *vs* C1-INH function measured in the same sample. The values are the mean of three independent experiments.

The discrepancy between C1-INH function and activity in the plasma of patient T.M. was suggestive of a type 2 mutation, so the presence of abnormal conformers of C1-INH was checked by plasma SDS-polyacrylamide gel electrophoresis (SDS-PAGE) and immuno-blotting. Non reducing Western blot analysis of plasma C1-INH showed a single band in normal pooled plasma, corresponding to native C1-INH (105 kd), 2 bands, around 250 and 96 kd, in the plasma of the individual homozygote for the Arg378Cys substitution (kindly provided by Dr. Margarita López-Trascasa, Unidad de Inmunología, Hospital Universitario La Paz/IdiPAZ, Madrid, Spain), and all the 3 bands in the plasma of our patient. (Fig. 2a). The 96 kd band was already observed in the study by López-Trascasa’s group, and was designated as a “latent-like” conformer^14^.

The 3 bands detected in samples of plasma with different C1-INH functional levels, were thus quantified by the use of a secondary antibody (Ab) conjugated with a fluorescent dye. C1-INH function positively correlates with the amount of the “native-like” band, and inversely correlates with the “non-native” bands (Fig. 2b). Thus, C1-INH antigen and function vary in the plasma of patient T.M., and this phenomenon associates primarily with fluctuation of the native-like form, while the other conformers of the protein seem to have more subtle variations. Actually, we cannot exclude a faster plasma clearance of the non-native C1-INH conformers^26^.

**Figure 3:**
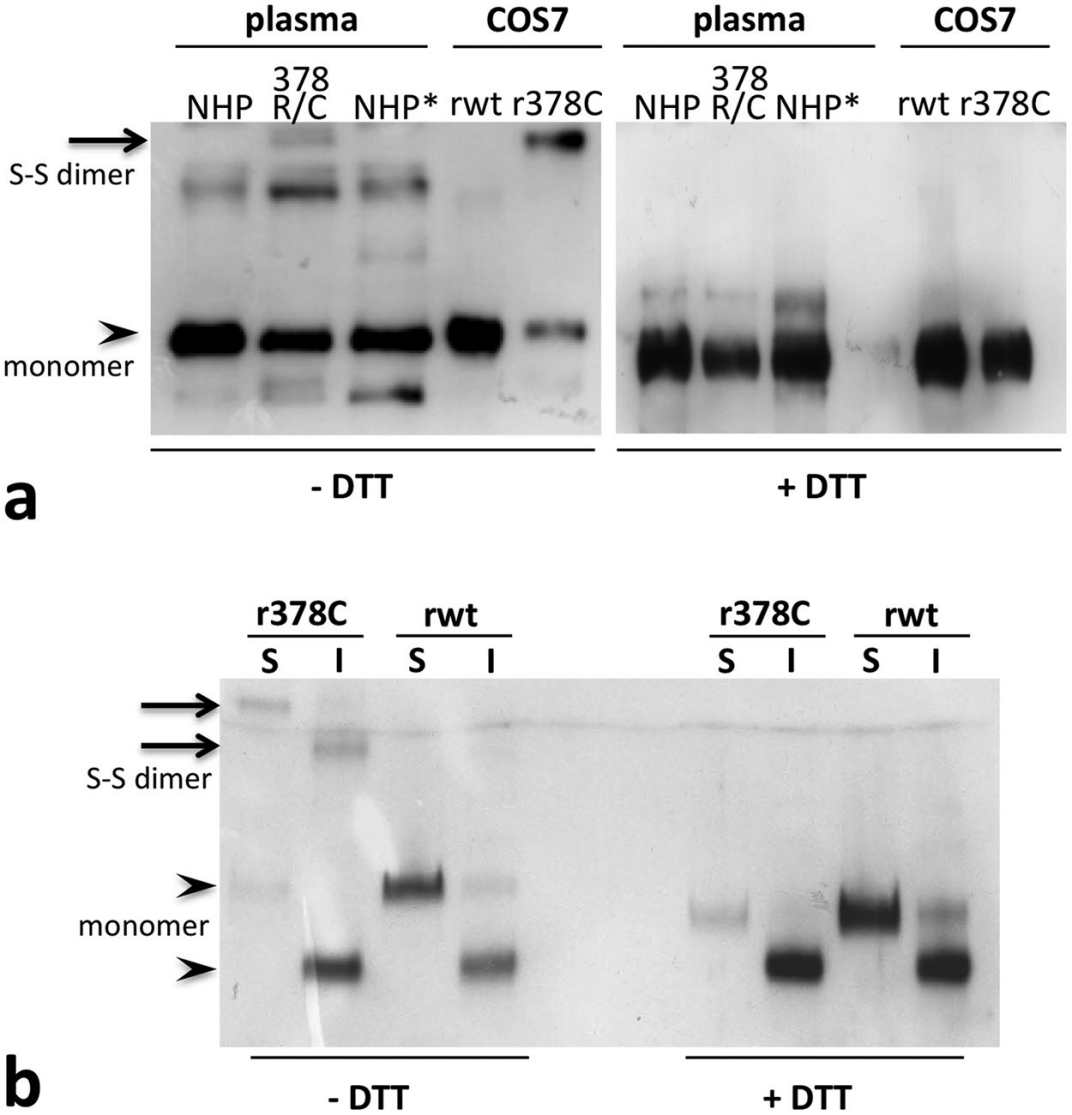
C1‐INH conformers in reducing and non‐reducing SDS-PAGE. (**a**) Plasma samples (NHP and 378 R/C) and recombinant C1-INH expressed in COS7 cells (rwt for the wild‐type construct and r378C for the mutant) were electrophoresed in reducing (+DTT, right) and not reducing (−DTT, left) SDS‐PAGE before blotting. When the disulphide bonds were reduced the higher bands disappeared. Normal human plasma (NHP) and kaolin activate plasma (NHP*) are shown as reference. (**b**) Recombinant C1‐INH present in cell media (S: secreted) or in the cell post‐nuclear supernatant (I: intracellular) were electrophoresed in reducing (right) and not reducing (left) SDS‐PAGE before blotting. Bands corresponding to monomeric (105 kd for the secreted form, slightly less for the intracellular, not fully glycosylated, form) and dimeric (around 250 kd) forms were indicated with arrowheads and arrows respectively. The primary Ab used was a commercial polyclonal antibody against C1‐INH (The Binding Site Ltd, Birmingham, UK).

To better characterise the “non-native” bands, we tested plasma C1-INH via SDS-PAGE in reducing and not reducing condition, including recombinant C1-INH obtained in COS-7 cells (fibroblast-like cell line derived from monkey kidney tissue) for comparison (Fig. 3). In non-reducing conditions, the higher band was clearly visible both in patient plasma and in the supernatant of COS cells expressing the Arg378Cys mutant, while it disappeared in the presence of the reducing agent (Fig. 3a), suggesting that the high molecular weight conformer is a disulphide-linked dimer (Fig. 1c). The dimers were already present inside the cells (Fig. 3b), meaning that they form in the oxidizing environment of the endoplasmic reticulum (ER).

We thus tested the plasma against conformation-specific antibodies. KII and KOK12 monoclonal antibodies (mAb), that recognize neoepitopes exposed on complexed and cleaved C1-INH, were used as primary Ab in Western blot after native-PAGE (data not shown). The dimer was not detected by these mAbs, suggesting that the disulphide bridge occurs between intact molecules. Next, we also tested the reactivity towards an in-house chicken polyclonal antibody targeted to the last C-terminal residues of the intact molecule, not present in the cleaved form. While the dimer was visualized, as expected, the 96-kd band seen in the plasma of the homozygous subject and patient T.M. was not detected by this Ab (supplementary Figure 2), suggesting that it, at least in part, might correspond to cleaved C1-INH. High amounts of cleaved C1-INH have already been reported in plasma of type 2 C1-INH-HAE subjects^27^. We cannot exclude that C1-INH is partly degraded due to experimental handling, as it may happen in C1-INH-deficient plasma.

**Figure 4:**
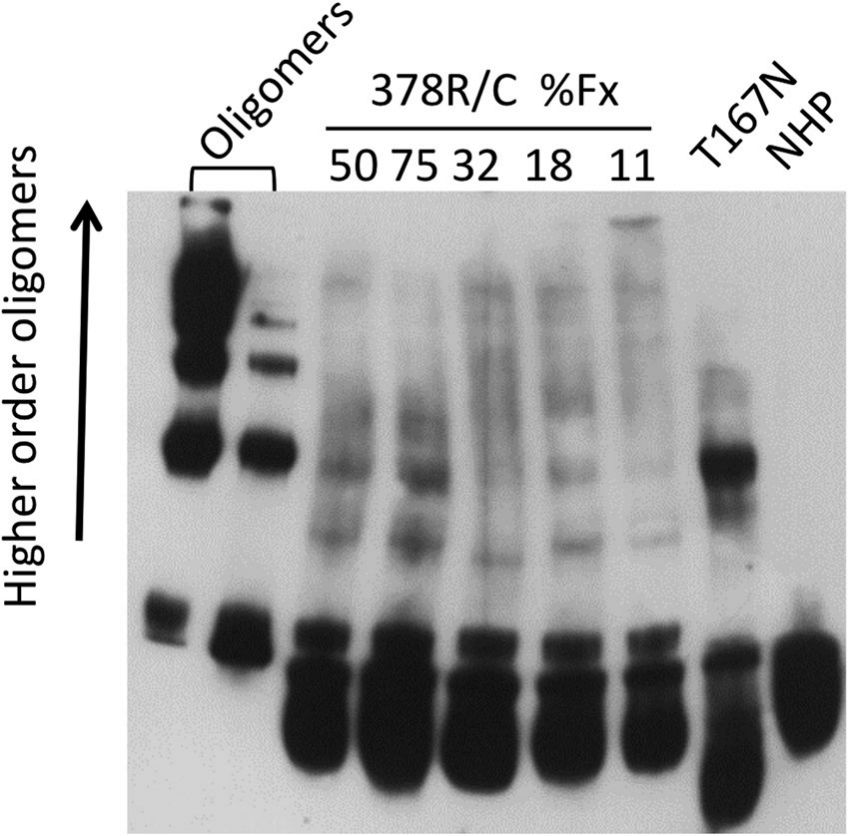
C1-INH oligomers in plasma. Non-denaturing PAGE and immuno-blot analysis of plasma from patient T.M. (378 R/C) at different functional values of C1-INH. Equal plasma volumes were loaded in each line. Heat-treated plasma C1-INH (oligomer), normal human plasma (NHP) and the plasma of a patient known to have circulating oligomers (T167N)^23^ are shown as reference. The primary Ab used was the 12-27-15 monoclonal antibody, revealed with a secondary Ab designed for chemiluminescent detection.

Finally, in order to check for the occurrence of circulating polymers, fresh citrated plasma was electrophoresed in non-denaturing PAGE followed by immune-blot analysis with the 12-27-15 mAb, that preferentially recognizes the polymeric conformation^23^ (Fig. 4). Although the monomer was the principal species present in all the samples, in patient’s plasma higher molecular weight bands were clearly visible, suggesting oligomers formation. These bands were not present in plasma from healthy controls. Reference polymers were obtained by incubating plasma-purified C1-INH at 55 °C for 30 minutes.

### Effects of the Arg378Cys mutation on protein expression

**Figure 5:**
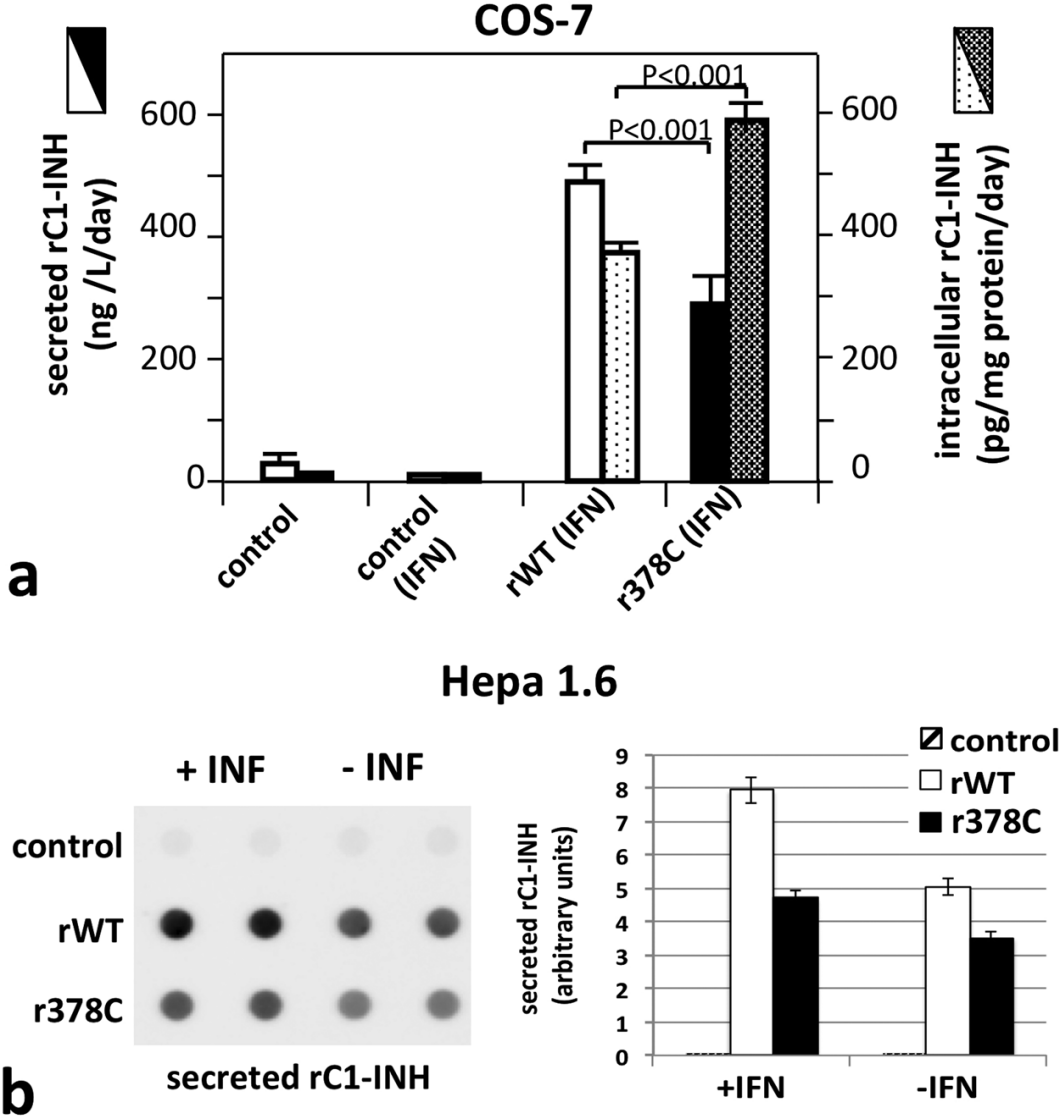
Expression of recombinant C1-INH. (**a**) Evaluation of secreted and intracellular rC1-INH in COS-7 measured by ELISA. Transfected COS-7 cell cultures were investigated for C1-INH concentration in supernatants (full bars) and in lysates (dotted bars), for non-transfected (control) and transfected cells (rWT and r378C for wild-type and Arg378Cys minigene plasmid, respectively). The activation in the presence of 1,000 U/mL interferon-γ is indicated (IFN). *P* values were calculated from the unpaired Student’s *t* tests. (**b**) Evaluation of secreted rC1-INH in Hepa 1.6 measured by Dot Blot. Transfected Hepa 1.6 cell cultures were investigated for C1-INH concentration in supernatants with and without IFN-γ for wild-type (open bars) and Arg378Cys (closed bars). On the left a representative Dot Blot is shown.

To assess if the Arg378Cys C1-INH has secretion defects, we tested its expression in different cellular models. COS-7 cells were transfected with the C1-INH minigene^28^ and recombinant C1-INH (rC1-INH) expression was induced through interferon gamma (IFN-**γ)** stimulation. In these conditions, the yield of the wild type protein in the culture supernatant almost doubled the amount of the Arg378Cys mutant. An inverse situation was observed after rC1-INH measurement in the post-nuclear supernatants (Fig. 5a). The same experiments were repeated in Hepa 1.6 cells, a murine liver-derived cellular model previously used to characterise other serpin deficiencies^29^. In resting conditions the ratio of wild-type versus mutated rC1-INH diminished, while under IFN-γ stimulation, the results were comparable with that obtained in COS cells, suggesting a lower secretion of Arg378Cys C1-INH at the higher expression levels induced by IFN-γ (Fig. 5b). Endogenous C1-INH was not detected spontaneously in supernatants or cell lysates in both resting and IFN-γ-activated cultures.

In *P*. *pastoris*, an expression system designed to potentiate heterologous protein expression, the difference in the yield of secreted wild-type versus mutated rC1-INH highly increased. Single colonies (15) were selected and grown in glycerol until OD_600nm_ was between 2 and 6 and then for 3 days in buffered complex medium with methanol (0.5%, v/v) to activate rC1-INH transcription. The culture media and the cell pellets were tested for secreted and intracellular rC1-INH by enzyme-linked immunosorbent assay (ELISA). The mean extracellular values were 2.1 ± 0.3 μg and 24.6 ± 4.4 μg per ml of culture medium for secreted Arg378Cys and wild-type respectively (p < 0.0001); and 0.20 ± 0.05 μg and 0.53 ± 0.18 μg per ml of culture for intracellular Arg378Cys and wild-type, respectively (p = 0.047) (Supplementary Figure 1). Loss of polymers in the pellet due to vigorous extraction procedures could be a cause of the low yield of intracellular Arg378Cys rC1-INH.

Altogether, the expression experiments suggest that the Arg378Cys C1-INH has a secretory defect that is exacerbated when protein synthesis is rushed, and cellular crowding augments.

### Effects of the Arg378Cys mutation on protein function

Both wild-type and Arg378Cys recombinant proteins formed stable complexes with their target proteases C1s and kallikrein, as indicated by SDS-PAGE (data not shown).

**Figure 6:**
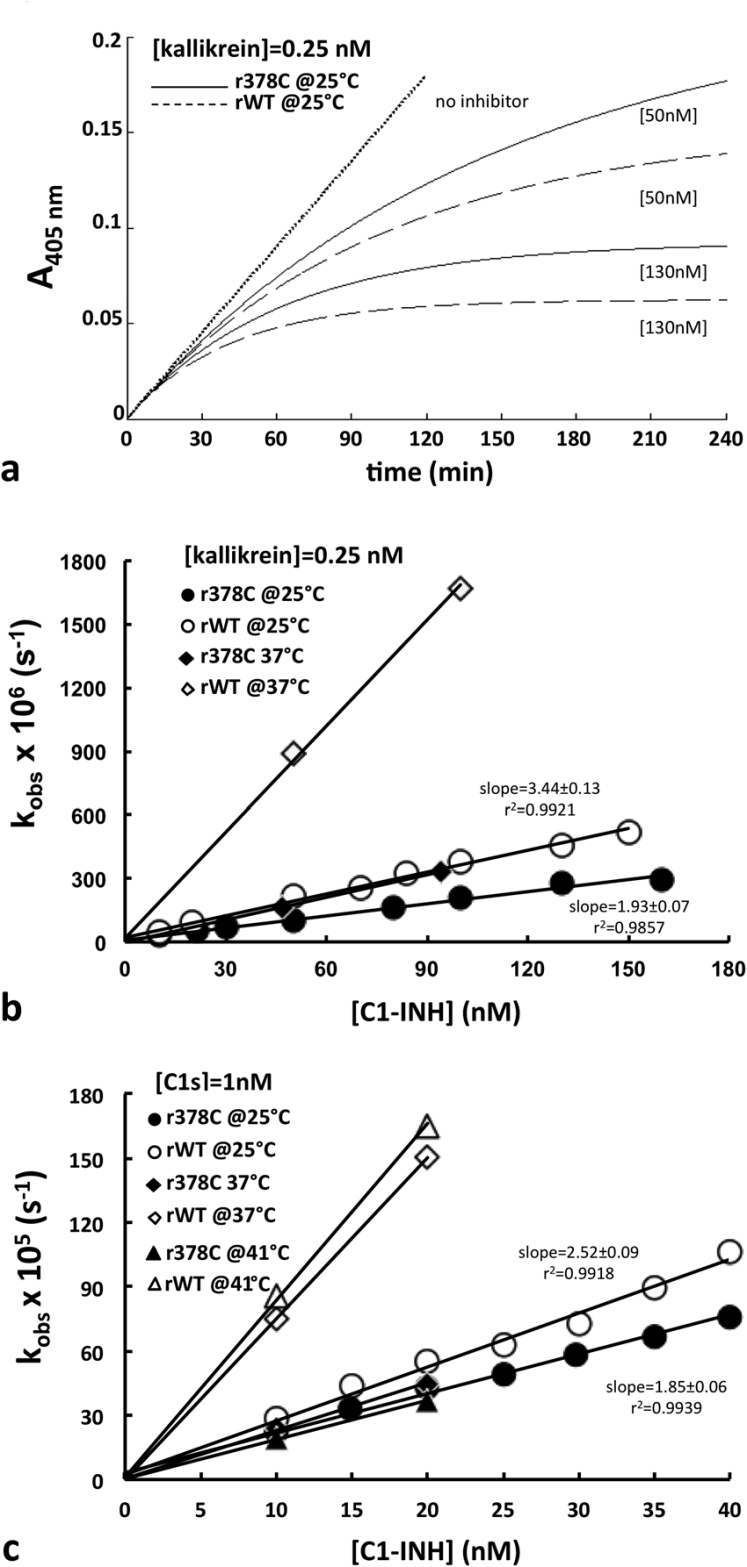
Protease inhibition assay. (**a**) Progress curve of the reaction of wild-type rC1-INH (dashed line) and Arg378Cys mutant (solid line) with kallikrein. (**b**) Plot of *k*_obs_ of C1-INH wild-type (open circle) and Arg378Cys (closed circle) *versus* [*I*]_0_ for kallikrein (**c**) and C1s. rC1-INH, substrate and protease were combined in a 2-ml cuvette and substrate conversion was recorded at 405 nm. Reagent concentrations are shown in supplementary Table I. Experiments were performed at 25 °C in 50 mM Tris, 10 mM Na_2_HPO_4_, 150 mM NaCl, 0.1% Tween pH 8.

Kinetics of inhibition analysis were set up under pseudo-first-order conditions, as summarized in Supplementary Table 1. Progress curves for rC1-INH with C1s and kallikrein were determined (Fig. 6a is given as an example).

Nonlinear regression analysis resulted in the values for *k*_obs_, *v*_0_ and *v*_s_ (see Methods). At 25 °C different rC1-INH concentrations were tested and the plot of *k*_obs_ versus the inhibitor concentration resulted in a straight line (Fig. 6b,c) indicating second order conditions. Accordingly in all the reactions *v*_0_ was always equal to the value obtained from controls without inhibitor (data not shown) and *v*_s_ was always zero (irreversible inhibitory reaction). The apparent second-order rate constants of inhibition (k_inh_) at 25 °C, calculated from Equation 2 as described in the Methods, were 2.95 ± 0.09 × 10^4^ M^−1 ^s^−1^ in the case of the Arg378Cys mutant and 4.03 ± 0.15 × 10^4^ M^−1 ^s^−1^ in the case of wild-type for C1s, 6.67 ± 0.39 × 10^4^ M^−1 ^s^−1^ and 11.52 ± 0.5 × 10^4^ M^−1 ^s^−1^ respectively for kallikrein (supplementary Table 2). Thus the ratio of k_inh_ of wild-type over Arg378Cys mutant at 25 °C is less than two for both proteases, with a higher activity drop for kallikrein (44%) than C1s (27%), in accordance to published data^14,30^. Indeed, SDS-PAGE analysis of the stoichiometry of inhibition (SI) showed that Arg378Cys rC1-INH required almost a 2-fold higher stoichiometric excess over kallikrein than wild-type to achieve full inhibition (data not shown). This observation suggests that the reduction in protease control by the mutant is mostly due to the unproductive turnover of the inhibitor, resulting in the release of a RCL-cleaved serpin and an active protease (as suggested by Western Blot analysis of plasma), and partly due to a non-optimal presentation of the RCL (see Fig. 1b for a description of the inhibition mechanism).

At higher temperature, 37 °C and 41 °C, the rise in activity of the wild-type is evident, whereas the activity of the mutant does not augment.

### Effects of the Arg378Cys mutation on protein stability

**Figure 7:**
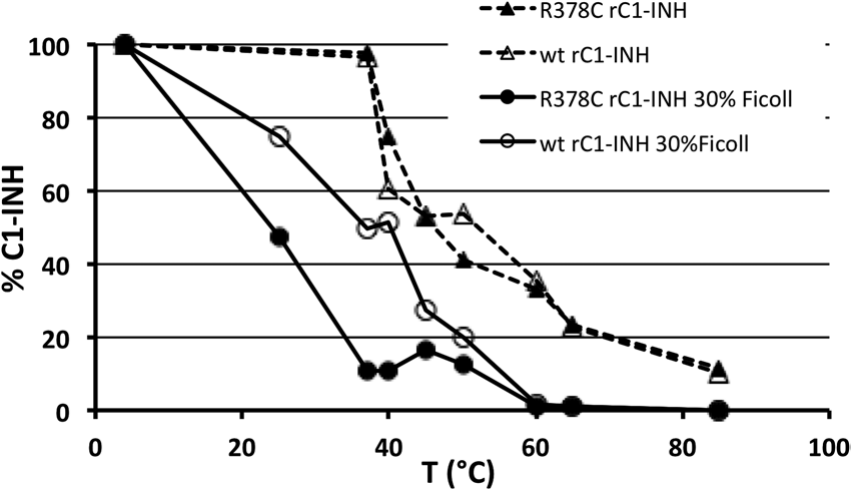
Effect of crowding on heat stability of rC1-INH. 250 μg/ml C1-INH wild-type (wt, open symbols) and Arg378Cys (R378C, closed symbols) were incubated for 2 hours at various temperatures with (circles) or without (triangles) 30% Ficoll. Samples were centrifuged at 10,000 × *g* for 20 min, concentration of C1-INH antigen was measured by ELISA and expressed as the percentage of antigen concentration compared to samples kept at 4 °C. Each point was assessed at least in triplicate.

Thermal denaturation assay is accepted as a reliable method to measure serpin conformation. At high temperatures, normal serpins denature and multimerize, this event causes a reduction of epitopes detected by polyclonal antibodies with an apparent reduction of C1-INH concentration^21^. Heat stability of Arg378Cys rC1-INH was compared with that of wild-type. Detectable antigen levels remaining in solution after two-hour incubation at different temperatures are shown in Fig. 7. In the 0.1% Phosphate Buffer Saline (PBS) the two proteins exhibited a similar thermal denaturation profile with indistinguishable curves. Since the degree of environmental “molecular crowding” has a relevant influence on protein denaturation and on their tendency to associate^31,32^, we performed the experiment in the presence of 30% Ficoll, a neutral, highly branched, high-mass, hydrophilic polysaccharide in a concentration likely reproducing intracellular macromolecular crowding^33^. In this condition, compared to wild-type rC1-INH, the Arg378Cys mutant displayed significantly lower heat stability, indicating a higher susceptibility to environmental conditions of the mutant protein.

**Figure 8:**
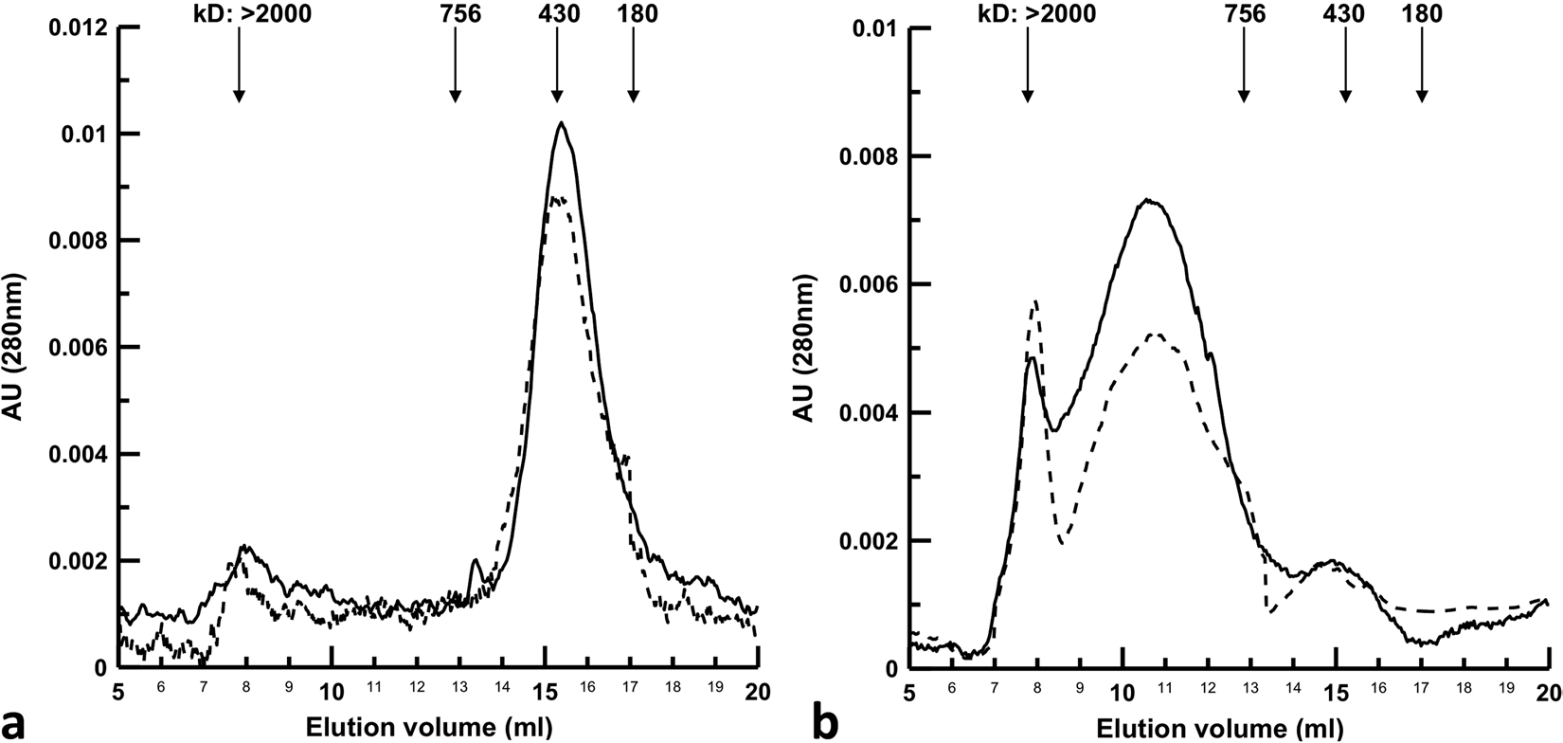
Gel filtration of native and thermally denatured rC1-INH. 25 μl of 2 mg/ml wild-type rC1-INH (dashed line) and Arg378Cys mutant (solid line) were applied to a Superose6^®^ column without pre-incubation (**a**), and with pre-incubation at 55 °C (**b**). The elution was continuously monitored at 280 nm. The arrows indicate the elution volume of marker proteins, from left to right: blue dextran 2,000, thyroglobulin (M_r_ 756 kd), ferritin (M_r_ 430 kd), catalase (M_r_ 213 kd).

To determine the protein state of aggregation and their propensity to form polymers, gel filtration experiments were performed: recombinant wild-type and Arg378Cys mutant were run on a Superose^®^ matrix before and after one-hour incubation at 55 °C (a temperature slightly above the calculated melting temperature) (Fig. 8a,b). Under both conditions, the recombinant proteins had a parallel elution profile. Without pre-incubation at high temperature the main peaks eluted at a volume corresponding to the hydrodynamic radius of ferritin (430 kd), and were identical to the monomeric C1-INH hydrodynamic size previously reported^34^ (Fig. 8a). After incubation at 55 °C the main protein fractions eluted between the column void volume (>2,000 kd) and 756 kd (Fig. 8b), indicating that low order oligomers were mainly formed. This is more evident in the case of the Arg378Cys mutant. These results were confirmed with plasma C1-INH (data not shown). This observation is consistent with the presence of oligomers in patient T.M. plasma (Fig. 4). A relevant fraction remained that eluted as a monomer, indicating either a cleaved or a latent form.

## Discussion

Mutation g.19332 C > T in *SERPING1*, which changes the arginine 378 into a cysteine, was described in a case of homozygous C1-inhibitor deficiency^14^. The propositus had low plasma level of C1-INH and suffered from severe recurrent angioedema. Heterozygous relatives also had significant reduction of C1-INH, but none of them had angioedema symptoms. The authors provide some insights on the biochemical function of the Arg378Cys mutation, suggesting that substitution at position 378 alters C1-INH specificity. Here we present a patient heterozygous for the same mutation, with abdominal symptoms that are not typical for HAE and no cutaneous or laryngeal angioedema symptoms. Her C1-INH and C4 levels, systematically tested, ranged from deep depletion to normal values (Supplementary Figure 1). We proved that the Arg378Cys mutant has an increased sensitivity to mild stress, which would explain this peculiar intermittency. Slight and temporary changes in environmental conditions inside the productive cells could promote Arg378Cys mutant misfolding, causing decreased secretion and low C1-INH plasma level. Indeed, some molecules will fold along the entire folding pathway leading to the desired functional monomers that, finally, circulate into the plasma as active protein inhibitors.

The Arg378Cys C1-INH displays a reduced control capacity of target proteases when compared to the wild-type, in agreement with the slight discrepancy observed between the antigenic and functional protein levels in the patient’s plasma. The alteration amplifies with temperature, providing first evidence that the Arg378Cys mutation renders the inhibitor sensitive to moderate stress conditions. In a purified system at 25 °C, the decrease in the constant of inhibition was around 25% toward C1s and 45% toward kallikrein; at 37 °C the activity diminished by 70% and 80% respectively and at 41 °C the drop was even higher. Indeed, the inhibitory activity of the mutant did not increase with temperature as that of the wild-type, as if the inhibitor acquired a conformation with altered inhibitory activity at high temperatures (Fig. 6). This is in line with recent data where Z alfa1-antitrypsin (AAT) mutation induces an equilibrium between a native inhibitory conformation and an aberrant, not completely folded, conformation^35^.

Our cellular studies demonstrate that the yield of secreted Arg378Cys C1-INH is rather reduced compared to that of the wild-type protein (Fig. 5), and such a difference is amplified by increased rates of protein synthesis. A secretory defect appears as the more likely mechanism leading to this impairment. A defective transcription was excluded, given that C1-INH mRNA in patient blood as well as in the recombinant cell lines is even higher than in the controls. Moreover, we observed an intracellulat accumulation of the mutant rC1-INH expressed in COS7 cells. Additionally, degradation or aggregation of the secreted Arg378Cys protein in culture media might contribute to its low extracellular concentration, but it is unlikely its main cause, since wild-type and Arg378Cys C1-INH exhibited similar electrophoretic pattern and gel filtration profile at the temperature used for cell growth. Complete or partial intracellular C1-INH retention has already been demonstrated with other C1-INH mutants^24^, as well as in genetic deficiencies of serpins, whose related diseases are collectively identified as serpinopathies^36^.

The underlying molecular mechanism of these diseases is the direct effect of a proneness to misfold due to the metastability of the native conformation. Indeed, unlike the majority of proteins, serpins fold into a metastable conformation (Fig. 1a) so that the highly favourable energetics for the formation of the cleaved-like conformation allows their unique mechanism of inhibition (Fig. 1b). An adverse consequence of the necessity to fold along a precise pathway to reach a metastable state is a risky folding landscape in which mutations can induce the accumulation of polymerization-prone folding intermediates. Actually, many natural serpin mutants have been described to promote the formation of long ordered polymers, which are more stable than the native metastable form. Polymerization can occur spontaneously or in combination with environmental factors. Long polymers cannot be properly channelled through the secretory pathways, resulting in extracellular protein deficiency. Examples of C1-INH oligomerization associated to impaired secretion have already been reported^21,22^. Unlike the variant described here, however, these mutants result in a constant level of plasma protein. Indeed, when we tested the plasma of patients bearing these polymerizing mutations for the presence of circulating polymers, none have been found (data not shown); conversely we did find C1-INH oligomers in the plasma of patient T.M., indicating that Arg378Cys C1-INH is mildly polymerogenic. Whereas long polymers are retained intracellularly, short serpin oligomers may escape and be secreted^37–39^. Gel filtration data of recombinant proteins corroborate this hypothesis, showing that Arg378Cys C1-INH preferentially forms short oligomers (Fig. 8). Similarly Z AAT oligomers have been found in patients’ plasma and the Z mutation has been proposed to be associated to the formation of monomers and oligomers other than higher-order polymers in the ER of hepatocytes^40^. The common pathological Z mutation of AAT causes the substitution of Glu342 with a Lys (mature AAT numbering). In the native protein the negative charge of Glu342 at the top of s5A salt bridges with the positive charge of Lys290 in the “bridge region”. Glu342 in AAT corresponds to Glu429 in C1-INH, and Lys290 to the mutated Arg378, thus the interaction between the two charged residues is equally broken in both proteins (Fig. 1a). Contrarily to Z AAT, however, C1-INH-HAE has never been associated to liver disease due to polymers accumulation inside the hepatocytes. C1-INH is synthesized in the liver at one-tenth the amount of AAT, so that the likely aggregation associated with the polymerization-prone mutations would be insufficient to menace the viability of the hepatocytes.

A gain of function of the circulating serpin polymers has been supposed, but their effect has so far remained elusive^38,41,42^. The presence of C1-INH polymers in the plasma of two patients suffering from type 1 C1-INH-HAE has already been reported^23^. The authors speculated that polymers can play a role as inflammatory mediators through the FXII-dependent kallikrein-kinin system activation, since they proved their ability to facilitate FXII activation *in vitro*^41^.

If the Arg378Cys mutation renders C1-INH prone to polymerization, it would not be expected that the same mutation has minimal consequences on the thermodynamic stability of the molecule, as seen in thermal denaturation experiments in phosphate buffer saline (Fig. 7, triangles). This is allowable when considering that ER polymers form during folding, whereas polymers produced with increasing temperatures come from already folded, native serpins. It is currently not clear whether the two processes follow the same mechanism. Notably, when tested in 30% Ficoll, a concentration likely reproducing intracellular macromolecular crowding^33^, the Arg378Cys mutant was much more prone to oligomerization than the wild-type (Fig. 7, circles). Both experiments and theory support the view that crowding influences protein association and conformation^43,44^, favouring all association processes, as well as influencing the folding and unfolding pathways, thus making possible that in our experiment both mechanisms of polymer formation occur simultaneously. Accordingly, the highly non-linear nature of the crowding phenomenon might favour the tendency of proteins produced within the crowded intracellular environment to acquire a misfolded conformation in response to small changes in cellular hydration, temperature, pH, etc.

In the proposita, the defect associated with the altered folding is exacerbated by the nature of the mutation: indeed, in the oxidizing environment of the ER, the exposed new Cys gives rise to non-functional homodimers through the formation of inter-molecular disulphide bonds. These conformers would not be able to inhibit proteases but could acquire new unexpected functions. There is now increasing evidence that the conformationally inactive forms of the serpins can assume some signalling tasks^42,45^. In particular, another disulphide-bonded dimeric form of a mutant serpin, antithrombin-III, was found in the plasma of individuals displaying severe venous thrombosis^46^. The authors suggested that the secreted dimers could acquire new prothrombotic functions. Moreover, the thiol-dependent quality control of the ER could mediate retention of this mutant protein harbouring an exposed cysteine residue. Anti-oxidant strategies could therefore be useful as a complementary therapeutic strategy for these patients, as it has been demonstrated for AAT mutants forming aberrant inter- and intra-molecular disulphide bonds involving an acquired Cys^47,48^.

The very low plasma levels of C1-INH measured intermittently, instead of at least the 50% expected from the wild-type allele, could be attributed to a higher catabolism of normal C1-INH due to the on-going activation of the proteolytic systems controlled by the deficient serpin, as previously suggested for C1-INH-HAE^27,49,50^. In one of the milestone papers on this subject, more than 30 years ago, the authors stated that their studies clearly showed that the actual situation is more complex and that both increased catabolism, predicted by the mechanism postulated above, and decreased synthesis of normal C1-INH contribute to the low serum concentration of this protein in all forms of HAE^50^. It has been difficult and unsuccessful to demonstrate a general mechanism of decreased circulating C1-INH; here we speculate that the unstable monomers, possibly folding intermediates, formed from Arg378Cys C1-INH, as well as from other conformational mutations, could behave as an infective seed recruiting wild-type monomers into the growing polymers, thus sequestering the wild-type protein from being secreted. The peculiarity of this mutant is that this does not happen constitutively, but under mild stress. An infective polymerization has also been demonstrated for antithrombin-III^51^ and AAT^48^ mutants, and is at the basis of the mechanism of aggregation of prion proteins that lead to spongiform encephalopathy, and other conformational diseases^52^.

There is ample evidence for involvement of the contact system in HAE attacks^53^. Yet, the critical threshold in the control of factor XIIa and kallikrein, that will impede profound activation of the contact system upon the generation of minute amounts of FXIIa, has not been established yet. In our patient C1-INH plasma levels are frequently in the ranges of those of patients presenting even severe clinical symptoms^54^. We cannot rule out the possibility that episodic abdominal symptoms reported by our patient could depend on angioedema of the gastrointestinal mucosa. However, their clinical characteristics make such a hypothesis very unlikely and the possibility that she was asymptomatic for angioedema as the other heterozygous carriers of this mutation our preferred conclusion. Present observation of the remittent angioedema phenotype of T.M. could ascribe a critical functional level of plasma C1-INH, and/or a critical structural configuration, to control contact phase enough to prevent from angioedema attacks. This scenario is reminiscent of the situation observed with the substitution therapy used as prophylaxis, where periodic C1-INH infusion protects the patient from angioedema attacks for a much longer time period than the drug half-life^55^. The reason for this long-term effect might lie in the fact that once the contact system is inhibited, through an adeguate amount of C1-INH in the physiological range, this takes time to reach a systemic activation and finally cause a localized attack.

To the best of our knowledge, this is the first report of a C1-INH mutant whose stability and functional activity appear to be finely tuned by environmental conditions (*i*.*e*. temperature, pH, oxidative stress), which could vary in situations of mild stress, such as hyperthermia or metabolic acidosis. The described observations provide a step forward towards the understanding of the molecular mechanisms making angioedema a phenomenon spatially- and temporally-confined^56^.

## Methods

### Subject

Patient T.M. is a 64-year old woman who has been followed in our C1-INH-HAE centre, located in the Luigi Sacco Hospital, from October 1996.

The patient gave written informed consent to participate to this study. All research and measurements adhered to the tenets of the Declaration of Helsinki, and this study was approved by the Ethics Committee of Luigi Sacco Hospital.

### Complement parameters testing

Blood samples were collected using sodium citrate as anticoagulant and plasma was stored at −80 °C until tested. C1-INH plasma function was measured with a commercially available chromogenic assay (Technocrome, Baxter-Immuno, Pisa, Italy). C1-INH and C4 antigens were measured using radial immunodiffusion plates (NOR-Partigen, Behring, Marburg, Germany). The complement profile was measured repeatedly on and off danazol treatment.

### Genetic studies

Genomic DNA was isolated from peripheral white blood cells and analysed by direct sequencing on an ABI Prism 310 Genetic Analyzer. All *SERPING1* (Ref.Seq NG_009625.1) exons and intron/exon boundaries were analysed in order to investigate the entire gene coding sequence.

Total RNA was obtained from whole blood with the QIAamp RNA Blood Midi Kit (Qiagen, Hilden, Germany), according to the manufacturer’s protocol. Real-time RT-PCR was performed with SYBR Green I using C1-INH-specific primers. Data were analysed with the relative quantification method and normalized to glyceraldehyde-3-phosphate dehydrogenase (GAPDH) expression.

### Immunoblotting of C1-INH in plasma

Plasma C1-INH was investigated by immunoblotting as described previously^57^. Briefly, 1.5 μl of pooled normal human plasma (NHP) and patients’ plasma were resolved by PAGE on 4–15% or 7.5% Mini-PROTEAN TGX precast gels (Bio-Rad Laboratories,). For native-Polyacrylamide Gel Electrophoresis (native-PAGE), Tris/Glycine running buffer was used and samples, after an initial 1:10 (v/v) dilution in PBS, were diluted 50% (v/v) in Native Sample Buffer (Bio-Rad). In the case of SDS-PAGE, the running buffer was supplemented with 10% SDS whereas PBS pre-diluted samples were subsequently diluted 25% (v/v) in 4X Laemmli Buffer before boiling for 5 minutes. Finally, when reducing conditions were wanted, dithiothreitol **(**DTT) was added to the sample buffer at a final concentration of 100 mM. Gels were blotted onto polyvinylidene fluoride (PVDF) membranes using the Trans-Blot Turbo Blotting System with Trans-Blot Transfer Pack consumables (Bio-Rad). Membranes were incubated with different primary antibodies depending on the information needed. KII and KOK12 mAbs (kindly provided by Dr. Diana Wouters, Department of Immunopathology, Sanquin Research, Amsterdam, The Netherlands) preferentially recognize neoepitopes exposed on inactivated and both complexed and inactivated C1-INH, respectively^58,59^. An in-house chicken polyclonal antibody designed against the last C-terminal residues of the intact molecule was used to discriminate the cleaved form. 12-27-15 monoclonal antibody (kindly provided by Dr. Daniel Elenius Madsen and Dr. Yaseelan Palarasah, Department of Cancer and Inflammation, Institute of Molecular Medicine, Faculty of Health Science, University of Southern Denmark, Odense, Denmark) was used in native-PAGE to detect C1-INH polymers^23^. Finally, non-conformational selective anti-C1-INH polyclonal antibodies (pAb) were used for comparison (a sheep pAb from The Binding Site Ltd, Birmingham, UK and a chicken pAb produced by our group). The immuno-complexes were revealed with secondary antibodies properly conjugated on the basis of the blot imaging strategy.

### Recombinant C1-INH expression in mammalian cells

The mutation Arg378Cys was introduced in the C1-INH minigene plasmid^28^ used for recombinant C1-INH expression.

COS-7 and Hepa1.6 cells (ATCC #1651 and #1830 respectively) were seeded in 10% fetal calf serum (FCS)-supplemented Dulbecco modified Eagle’s minimal essential medium (DMEM) +/− F12 medium, in 6-well plates (0.7 × 10^6^ cells/well) and transfected using the FuGene6^®^ method (1.5 µg DNA/3 µL reagent) (Roche Biochemicals, Meylan, France) according to the supplier’s protocol or with Polyethyleneimine ‘MAX’ (PEI) (Polysciences Europe, Germany), as previously described^60^. Six hours after transfection, the cells were treated with IFN-γ (1,000 U/mL) for 24 hours, then both cells and culture supernatants were collected. Post-nuclear cell supernatants were prepared in lysis buffer (0.5% NP40, 2 mM CaCl2, 1 µM leupeptin, 1 µM pepstatin, 1 mM phenylmethylsulfonyl fluoride **(**PMSF), 10 mM NaCl, 10 mM Tris-HCl, pH 7.5) eventually with 10mM N-Ethylmaleimide (NEM) supplementation, and subsequently centrifuged at 20,000 × *g* for 20 min to discard the nuclei pellet. Centrifugation at 10,000 × g was then preferred to avoid loss of polymers in the pellet. C1-INH expression levels were measured using ELISA or a quantitative dot-blot assay. Results were indicated in absolute concentration for the culture supernatants whereas for the cell lysates C1-INH levels were expressed relatively to the cell total protein content.

### Recombinant C1-INH expression in Pichia pastoris cells and purification

Human C1-INH cDNA was kindly provided by Dr. Erik C. Hack (Sanquin Research, Amsterdam, The Netherlands) and expressed in *Pichia pastoris* X-33 (Invitrogen Life Technologies) through the expression vector pPICZαA^61^.

For intracellular C1-INH quantification, cell lysates were prepared by adding 2.5 ml of CelLytic-Y reagent to each gram of yeast cell pellet. 4 grams of glass beads per gram of wet cell pellet were then added, and the suspension was vortexed several times, with 1-minute incubation on ice after each vortex. To pellet the cellular debris, the lysed cells were centrifuged for 10 minutes at 12,000 × g.

Recombinant C1-INH was purified from *P*. *pastoris* supernatant by cation exchange liquid chromatography on a MonoS 5/50 GL Tricorn column (GE Healthcare Life Science). Immediately after purification, the peak fractions were dialyzed against PBS containing 0.1% Polyethylene Glycol (PBS-0.1% PEG), concentrated and stored at −80 °C.

The recombinant C1-INH from *P*. *pastoris* was used to perform all the functional and structural studies detailed below.

### Gel electrophoresis

Integrity of recombinant C1-INH was assessed by 7.5% SDS-PAGE. Since recombinant C1-INH expressed in yeast appeared as a smear on SDS-PAGE due to the heterogeneous glycosylation of *P*. *pastoris* products, to obtain sharper bands *N*-linked oligosaccharides were removed by incubating with endoglycosidase H (New England Biolabs). After electrophoresis proteins were stained with the fluorescent dye SYPRO Ruby (Molecular Probes). Cleaved C1-INH was identified for its faster mobility on SDS-PAGE and quantified using a Typhoon 9200 laser scanner (GE Healthcare Life Science).

Bands composition was confirmed by western blotting.

### Complex formation with target proteases

Equimolar amounts (7 μM) of recombinant C1-INH and C1s or kallikrein were incubated for 1 hour at 37 °C in in 50 mM Tris, 10 mM Na_2_HPO_4_, 150 mM NaCl, 0.1% Tween pH 8. After incubation *N*-linked oligosaccharides were removed by endoglycosidase H (as above) and subjected to SDS-PAGE.

### Inhibition assays

The apparent second order rate constants of inhibition (*k*_inh_) were determined in the presence of chromogenic substrate by analysing the progress curves for the formation of *p*-nitroaniline. Each protease (E) was tested with a minimum of seven C1-INH (I) concentrations under pseudo-first order [I]_0_/[*E*]_0_ ratios (as detailed in supplementary Table 1).

Reactions were performed at 25 °C in 50 mM Tris, 10 mM Na_2_HPO_4_, 150 mM NaCl, 0.1% Tween pH 8. Buffer, inhibitor, and substrate were mixed in a 2-ml cuvette and reactions were initiated by addition of a fixed amount of protease. Product accumulation was continuously recorded by Cary 4E spectrophotometer (Varian, Inc) at *A*_405nm_ (A). A typical progress curve experiment consisted of 6 assays (1 zero and 5 nonzero concentrations of inhibitor). The progress curve data were fitted by nonlinear regression analysis to the equation describing the mechanism of slow binding inhibition (Equation 1)^62^. *k*_*obs*_, the observed first order inhibition rate constant in the presence of substrate, *v*_0_, the zero time value for the velocity of substrate hydrolysis, and *v*_s_, the steady state velocity at completion of the reaction, were obtained. Under second order conditions *k*_inh_ is derived from the plot of *k*_obs_ *versus* [*I*] using the relationship in Equation 2^63^, where S is the substrate for which I is an inhibitory analog and K_M_ the relative Michaelis constant.

1 $${\rm{A}}={{\rm{v}}}_{{\rm{s}}}{\rm{t}}+\frac{({{\rm{v}}}_{0}-{{\rm{v}}}_{{\rm{s}}})\,(1-{{\rm{e}}}^{-{{\rm{k}}}_{{\rm{obs}}}{\rm{t}}})}{{{\rm{k}}}_{{\rm{obs}}}}$$

2 $${{\rm{k}}}_{{\rm{obs}}}=\frac{{{\rm{k}}}_{{\rm{inh}}}{[{\rm{I}}]}_{0}}{(1+\frac{[{\rm{S}}]}{{{\rm{K}}}_{{\rm{M}}}})}$$

Each experiment was performed at least in triplicate.

### Thermal denaturation

Heat stability assays were performed according to Stein *et al*.^64^. Recombinant C1-INH proteins (250 μg/ml) were incubated for 2 hours at different temperatures in PBS-0.1% PEG, centrifuged for 20 min at 10,000 × g and quantified by ELISA. In order to reproduce C1-INH physiological environment, experiments were replicated in the presence of 30% Ficoll^65–67^.

### Gel filtration

Gel filtration was performed on a Superose matrix (10/300 GL Tricorn column, GE Healthcare Life Science) connected to a fast protein liquid chromatography system (GE Healthcare Life Science). 25 μl samples at the protein concentration of 2 mg/ml were loaded with a flow rate of 0.5 ml/min and the elution monitored at 280 nm. To test C1-INH oligomerization, prior to gel-filtration, samples were incubated at 55 °C and 70 °C for 1 hour.

### Molecular modelling

The model of the mutant was built on the basis of 1M6Q (Protein Data Bank accession number), an homology model of the serpinic domain of C1-INH^68^.

To build the dimer we used Rosetta online Server (ROSIE) to perform a local docking of two mutated molecules^69^. Among 1000 poses we chose the structure with the shortest distance between the cysteines. Then we performed a refinement of the structure allowing for loop adjustment using MODELLER v. 9.16^70^. A sulfur bridge patch was imposed between the mutated cysteine residues.

We added two complex glycans (a total of three are bound to the serpinic domain of C1-INH, http://www.uniprot.org/uniprot/P05155) to check for steric clashes. The virtual glycosylation was performed with the help of the Allosmod online server^71^ (https://modbase.compbio.ucsf.edu/allosmod/).

Molecular graphics were performed with the UCSF Chimera package. Chimera is developed by the Resource for Biocomputing, Visualization, and Informatics at the University of California, San Francisco (supported by NIGMS P41-GM103311)^72^.

### Data availability

The datasets generated during and/or analysed during the current study are available from the corresponding author on reasonable request.

## Electronic supplementary material

Supplementary data
