# Supplementary material for: Intermittent C1-Inhibitor Deficiency Associated with Recessive Inheritance: Functional and Structural Insight
Source: Sci Rep. 2018 Jan 17;8:977. doi: 10.1038/s41598-017-16667-w (PMC5772639; doi:10.1038/s41598-017-16667-w)
Supplement: Supplementary file 1 — Supplementary data [file 41598_2017_16667_MOESM1_ESM.pdf]

INTERMITTENT C1-INHIBITOR DEFICIENCY ASSOCIATED WITH RECESSIVE INHERITANCE: FUNCTIONAL AND STRUCTURAL INSIGHT

Sonia Caccia[a], Chiara Suffritti[a], Thomas Carzaniga[a], Romina Berardelli[b], Silvia Berra[a], Vincenzo Martorana[c], Anna Maria Fra[b], Christian Drouet[d], Marco Cicardi[a, e]

[a] "L. Sacco" Department of Biomedical and Clinical Sciences, University of Milan, via GB Grassi 74, 20157 Milan, Italy.

[b] Department of Molecular and Translational Medicine, University of Brescia, Italy.

[c] Institute of Biophysics, National Research Council of Italy, Palermo, Italy

[d] GREPI EA7408, Universite Grenoble Alpes, and CREAK, CHU Grenoble POBox 10217, 38043 Grenoble, France.

[e] Luigi Sacco Hospital, via GB Grassi 74, 20157 Milan, Italy.

* *Corresponding author*: Sonia Caccia, "L. Sacco" Department of Biomedical and Clinical Sciences, University of Milan, via GB Grassi 74, 20157 Milan, Italy.

Tel: +39-0250319664; e-mail: sonia.caccia@unimi.it

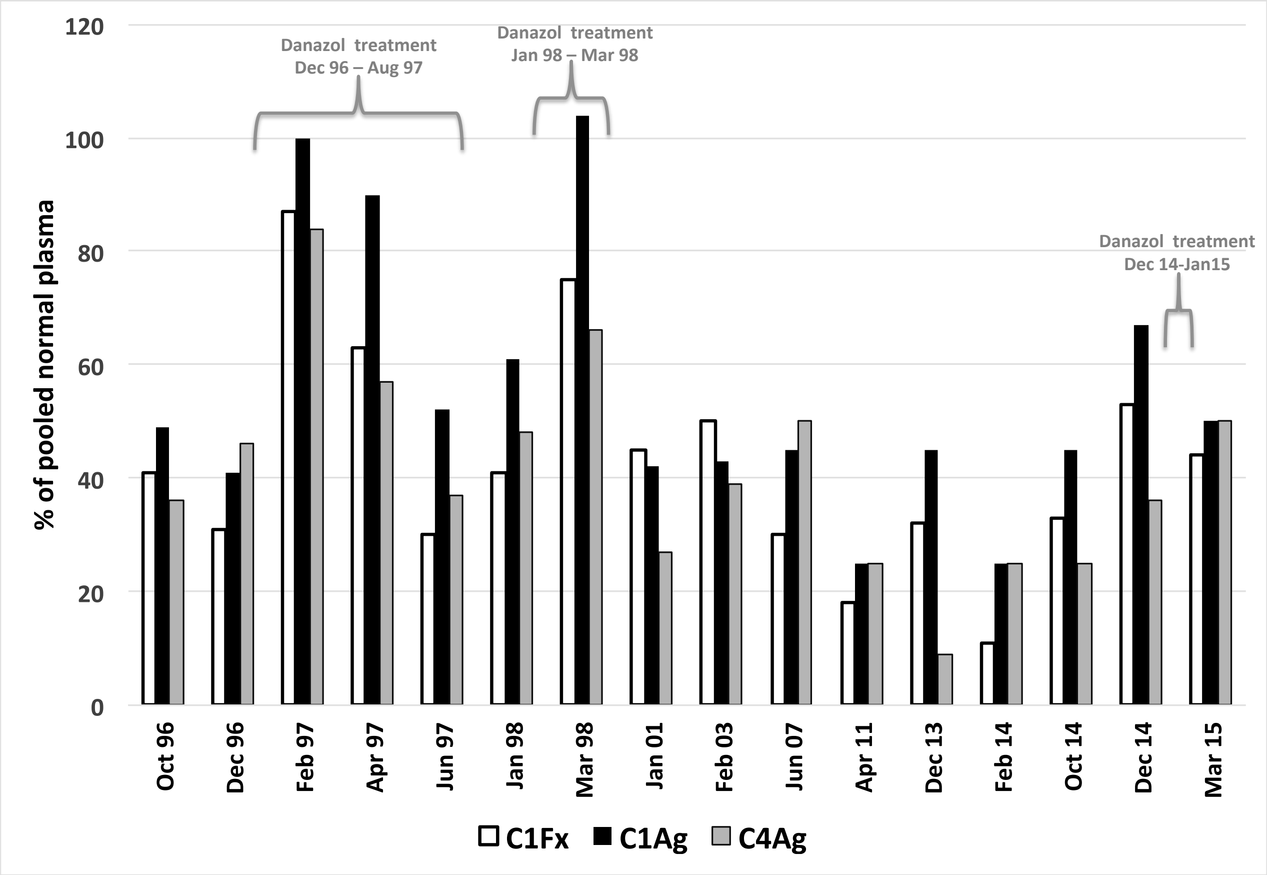

Supplementary figure 1. Complement parameters. Serial plasma levels of C1-INH function (white bars), C1-INH antigen (black bars) and C4 antigen (gray bars) in patient T.M. referred as a percentage of the pooled normal plasma used as reference.

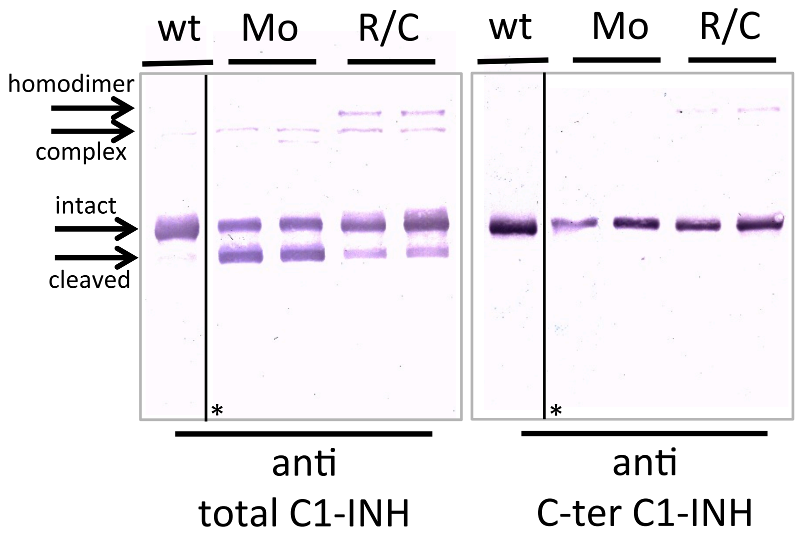

Supplementary figure 2. Analysis of the 96-kd band. SDS- PAGE and immuno-blot of plasma samples revealed with in house chicken polyclonal antibodies designed against the whole molecule (anti total C1-INH, left) or against the 10 C-terminal residues of the intact C1-INH (anti C-ter C1-INH, right), not reacting with the cleaved form. Normal human plasma (NHP), the plasma of two patients heterozygous for the C1-INH Mo mutation (Mo), which have already been reported to contain RCL cleaved C1-INH, and patient T.M. plasma with two different C1-INH concentrations (R/C) are analysed. Intervening lanes were removed for clarity, but the relative positions of the bands were unaltered. *The intervening lines were cut for clarity purposes.

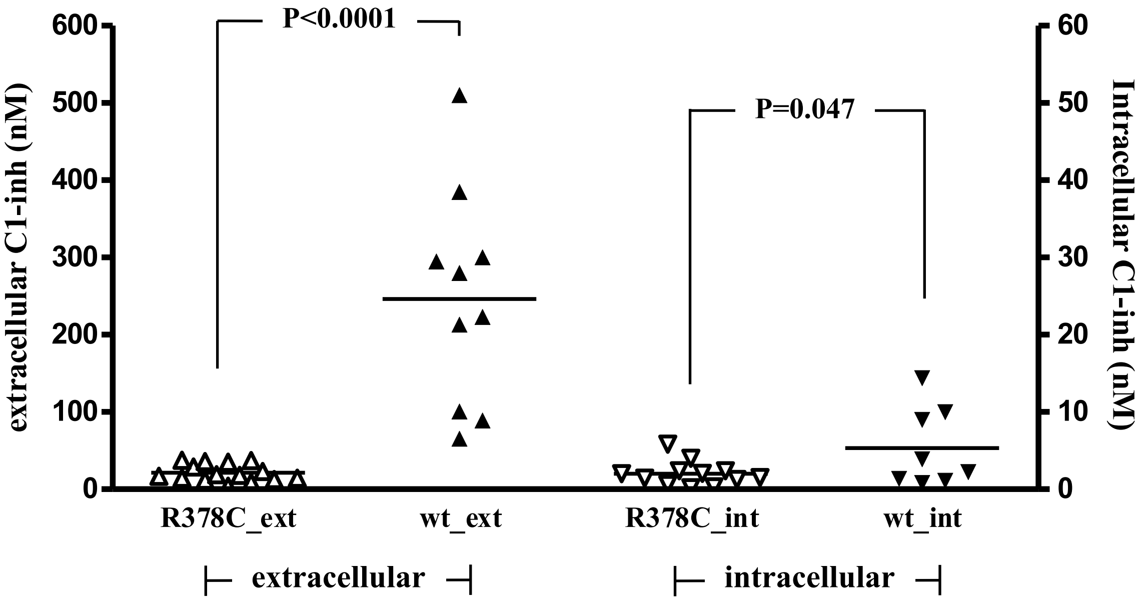

Supplementary figure 3. *Expression of C1-INH proteins in P. pastoris cells.* Single colonies (15) were selected and grown in glycerol until $OD_{600\ nm}$ was between 2 and 6 and then for 3 days in buffered complex medium with methanol (0.5%, v/v). The culture media and cell pellet were tested for secreted and intracellular C1-INH by means of ELISA. The mean extracellular values were 2.1 ± 0.3 µg/ml and 24.6 ± 4.4 µg/ml for secreted Arg378Cys and wild-type respectively (upwards triangles); and 0.20 ± 0.05 µg/ml and 0.53 ± 0.18 µg/ml for intracellular Arg378Cys and wild-type, respectively (downwards triangles). (WT and R378C for wild-type, open symbols, and Arg378Cys, closed symbols, respectively). P values from unpaired Student's t test are indicated.

Protease	[Protease] (nM)	Substrate	[Substrate] (μM)	[C1-Inh] (nM)
C1s	1	H-D-KGR-pNA	240	10-15-20-25-30-35-40
Kallikrein	0.025	H-D-PFR-pNA	250	10-20-30-50-80-130-160

Supplementary Table I. Reagent concentrations for kinetic assays.

Protease	Wild-type rhC1-Inh	R378C rhC1-Inh
	k_{inh} ($M^{-1}s^{-1}$)	k_{inh} ($M^{-1}s^{-1}$)
C1s	$(4.03 \pm 0.15) \times 10^4$	$(2.95 \pm 0.09) \times 10^4$
Kallikrein	$(11.52 \pm 0.50) \times 10^3$	$(6.67 \pm 0.39) \times 10^3$

Supplementary Table II. Apparent second-order rate constants of inhibition for the interaction between recombinant C1-INH and proteases.
